# Development and the art of nutritional maintenance

**DOI:** 10.1017/S0007114522001490

**Published:** 2022-09-14

**Authors:** David S. Gardner, Clint Gray

**Affiliations:** 1 School of Veterinary Medicine & Science, University of Nottingham, Sutton Bonington, LE12 5RD Loughborough, UK; 2 Gillies McIndoe Research Institute, Wellington, New Zealand; 3 University of Otago, Wellington, New Zealand

**Keywords:** Nutrition, Refined diet, Developmental Origins of Health and Disease, Salt, Fibre

## Abstract

Development from early conceptus to a complex, multi-cellular organism is a highly ordered process that is dependent on an adequate supply of nutrients. During this process, the pattern of organ growth is robust, driven by a genetic blueprint and matched to anticipated body mass with high precision and with built-in physiological reserve capacity. This apparent canalisation of the developmental process is particularly sensitive to variation in environmental stimuli, such as inappropriate drug or hormone exposure, or pattern of nutrient delivery. Significant variation in any of these factors can profoundly affect fetal and neonatal growth patterns, with later detriment for physiological function and/or reserve capacity of the resultant adult, with potential health impact. This paradigm shift in science has become known as the Developmental Origins of Health and Disease (DOHaD). Over the last 30 years, many animal and clinical studies have vastly expanded our fundamental knowledge of developmental biology, particularly in the context of later effects on health. In this horizons article, we discuss DOHaD through the lens of nutritional quality (e.g. micronutrient, amino acid, NSP intake). The concept of ‘*Quality’* was considered undefinable by Robert Persig in his book, ‘*Zen and the Art of Motorcycle Maintenance*’. Here, development and the art of nutritional maintenance will define quality in terms of the pattern of nutrient intake, the quality of development and how each interact to influence later health outcomes.

The human body has evolved over the last 250 000 years with a genetic blueprint honed to sustain life on diets similar to that of the Palaeolithic era^(1)^. The term Palaeolithic, meaning ‘of the age of stone’, is a period that covers the majority of human existence on earth. The Palaeolithic era extended from 2·5 million years ago to around 10 000 B.C.^(2)^ At this time, humans lived a predominantly nomadic, hunter-gatherer lifestyle until the Neolithic revolution around 10 000 years ago established agriculture, animal husbandry and a relatively stable supply of food throughout the year. Since that time, human bodies have evolved, genetically, little more; our metabolism remains Palaeolithic. From archaeological evidence, the early Palaeolithic diet had much more meat, fish, vegetables and fruits, when compared with a modern Western diet, with no refined nutrients^(3–5)^. Macronutrient composition was estimated to be protein (38 % of total energy), carbohydrate (23 % of total energy), fat (39 % of total energy) and a fibre intake of > 42·5 g/d, similar to the pattern of intake of modern hunter-gatherer societies. This contrasts markedly with modern Western diets (16 % protein, 49 % carbohydrate, 34 % fat, < 20 g fibre)^(6)^. Additionally, Palaeolithic diets had very little, to no, direct intake of ‘refined or added’ nutrients such as sugar and very little Na intake as ‘salt’ (∼256 mg/4184 kJ [1000 kcal]). Again, this contrasts with estimates of the current Western diet where sucrose may contribute up to 8 % of total calories (100 + g/d added sugar) and average added salt intake is ∼ 9–10 g/d^(1,7)^. Despite the unparalleled availability of good quality foods in the majority of Western countries, the increase in refined foods with high-energy density has led to a double burden of obesity and micronutrient malnutrition^(8)^. What determines food intake?

The demand for essential nutrients, those for which the body has dispensed with pathways necessary to form endogenously, drives intake of foods replete with those essential nutrients, which were presumably widely available in the environment of Palaeolithic man. It is why food intake is driven by the necessity to meet requirements for the essential amino acids (through intake of protein) and essential fatty acids (through intake of fat)^(9)^. There are no ‘essential’ carbohydrates despite dependence on glucose by the brain and erythrocytes. The essentiality of glucose is clear metabolically because the body expends significant energy retaining those pathways that can form sufficient carbohydrates to meet endogenous glucose demand, via hepatic and renal gluconeogenesis^(10)^. It is therefore axiomatic that for those extinct metabolic pathways, sufficient essential nutrients must be present in the environment to meet needs, assuming an appropriate diet pattern. For extant pathways, this is not the case, and the body invests energy in preserving functional redundancy to buffer short- or long-term fluctuations in supply. If the pattern of food intake is one where the intake of raw or unrefined, minimally processed seasonal foods is maximised, then requirements for other macronutrients such as fibre or essential vitamins and minerals can usually be met through consumption of these foods. Dietary dilution or modification of essential nutrients, as can happen with refined and processed foods that are high in energy but low in nutrients, encourages increased food intake^(11)^. Therefore, dietary advice for maximising nutritional maintenance should be relatively simple; focus should not be on maximising individual macronutrients such as protein and fat (e.g. the Atkins or ‘paleo’ diet) but on minimising intake of any ‘refined or processed’ macronutrients such as processed meat (refined protein), sugar (refined carbohydrate) and refined/processed fats (e.g. trans-fats). High intake of fruits, nuts, fish, vegetables, natural oils, whole grains, legumes and yogurt should be emphasised because such a pattern of food intake is, unsurprisingly, associated with beneficial health outcomes^(12,13)^. Minimising intake of refined food is also likely to limit intake of other minerals that when consumed in excess, can be detrimental to health, such as Na as salt^(14,15)^. High intake of any refined food and/or refined macronutrient is almost invariably associated with an increased incidence of non-communicable disease^(11,16–19)^.

Over the last decade, Raubenheimer and Simpson took a holistic approach to nutrient intake, proposing the geometric framework or ‘nutritional geometry’ to answer these fundamental questions of what represents a macro-nutritionally balanced diet and how this may be leveraged to optimise healthspan^(9,20)^. The state-space nutritional modelling method made measurable the interactive effects of dietary energy, protein, fat and carbohydrate on food intake, cardiometabolic phenotype and longevity in rodents^(21,22)^ and later confirmed in humans^(23)^. Such a framework as a read-out of drivers of food intake allowed for the principle mechanistic pathways to be elucidated such as the effect of essential, branched-chain amino acids on hepatic mitochondrial function and hepatic mammalian target of rapamycin activation. *Is maintenance of adult health therefore primarily about controlling food intake?*

Intake of sufficient essential nutrients is only one element of the overall balance of that specific nutrient. Using protein as an example, 35 g/d is approximately the minimum to maintain nitrogen balance^(24)^. The current average intake is approximately 85 g/d (Department of Health and FSA, 2004). The dynamic flux of protein turnover in the body, at a significant energy cost, is approximately 300–350 g/d^(25)^. Protein degradation approximately matches intake such that amino acid balance is achieved. Why invest so much energy in protein turnover, relative to intake/expenditure? Because it allows for precise metabolic control, such that individual amino acids may be partitioned to certain functions on-demand, and relatively quickly to facilitate immune defence, formation of blood cells, hormone production, muscle growth and repair. In the context of nutritional science, ‘read-outs’ of nutritional status of any individual by measuring single, spot samples of either intake of protein, the output of nitrogen or level of amino acids in blood will only reflect an individual’s nutritional status to a limited extent. Estimates of the rate function of protein or amino acid turnover, alongside a spot measurement of intake or plasma level, are required to fully assess adequacy of protein/amino acid nutrition. Taking recent examples where a measure of rate function has been estimated, using either stable isotopes^(26)^ or long-term highly controlled nutrition studies^(27)^ has revolutionised aspects of our basic understanding of how basal metabolism changes with age^(26)^, how our bodies handle salt^(27)^ or the turnover of fat cells^(28,29)^. In light of these data, textbooks will have to be re-written. Additionally, if energy demand (e.g. maintenance of protein turnover, balancing losses) drives food intake^(30)^, then the physical activity level has also to be considered. Physical activity by definition uses lean mass and therefore increases endogenous protein turnover to support the activity and repair any muscle microdamage. Greater demand matched by greater intake results in an increased rate of protein turnover, enabling greater metabolic control, that is, more efficient matching of intake to expenditure^(31)^. Integration of physical activity level into metabolism is integral to evolution of human metabolic control; loss or reduction in one (e.g. physical activity level) leads to loss or reduction in the other (e.g. metabolic control^(32)^).

For humans acculturated to a Westernised society, daily physical activity level has significantly reduced^(33)^, leading to a lesser ability to regulate energy turnover. This perturbed energy regulation, in combination with a greater ability to easily acquire energy-dense foodstuffs likely underpins the rise in non-communicable diseases such as obesity and type 2 diabetes^(32)^. In contrast, our ancestors would have engaged in physical activity during times of nutritional uncertainty to acquire food, increasing lipolysis and exhausting energy reserves as fat in adipose tissue. Indeed, relative to other great apes, human hunter gatherers expend more energy but less time on subsistence and therefore need substantially more energy per hour^(34)^. Kraft *et al.* suggest that such an expanded energy budget is met primarily by increasing rates of energy acquisition, rather than energy-saving adaptations, such as bipedalism or sophisticated tool use^(34)^. Yet now, our modern society is replete with energetically efficient, energy-saving adaptations and acquisition of excess energy requires little input beyond a short walk or drive. Such an imbalance in energy budget, relative to ancestral man, fuels excess energy intake relative to expenditure, with the small daily excess being stored, remaining unused, in adipose tissue. A daily excess of 100–200 kcal is hard to regulate, relative to the amount of stored fat (> 100 000 kcal). Thus, the ‘art of nutritional maintenance’ is to consume sufficient essential (micro) nutrients to support nutrient turnover optimally during the differing stages of life, during growth and development and during reproductive phases while supporting adequate maintenance/defence of the body for the remainder of the individuals healthspan. Broadly speaking, the refined, high-energy but micronutrient poor, Western diet represents a unique type of malnutrition to challenge optimal healthspan. The challenge for Developmental Origins of Health and Disease (DOHaD) science, and the delayed, temporal associations shown with health outcomes^(35,36)^ is therefore to identify which, and when, nutrient imbalances underpin such Western malnutrition and shorten healthspan by increasing the proportion of individuals with later non-communicable disease. Even if the data were available, what could be done to alter such a developmental trajectory?

## Development

For any healthy, adult female mammal, pregnancy and the products of reproduction will constitute the greatest anabolic (during gestation), then catabolic (during lactation) phase they will experience during their healthspan. Reproduction places unique demands on metabolic cycles and nutrient partitioning. During pregnancy, non-essential amino acids such as glycine become ‘conditionally essential’ due to high demand from growth of the products of conception. It has long been known by agriculturalists that inadequate (e.g. low crude protein) or unbalanced (e.g. insufficient glycine) nutrient intake during gestation and lactation impacted tangible outcomes such as rates of fetal or neonatal growth. These outcomes were easily recorded by measuring birth or current weight. In the post-war era, the emphasis was on maximising the efficiency of productive traits to support the nutritional health of the post-war population. Hence, much research that we now know as ‘developmental programming’ was indeed first elucidated, thanks largely to the collective efforts of agricultural scientists such as J Hammond^(37,38)^, LR Wallace^(39)^ and Robert McCance & Elsie Widdowson^(40–43)^. The latter pairing laid down the scientific foundations of DOHaD, outlining a mechanistic paradigm for how nutrition was able to impact current and future phenotype. The very first issue of The Proceedings of the Nutrition Society included two papers reporting a role for nutrition during pregnancy or lactation on later developmental outcomes^(44,45)^. Nevertheless, David Barker and Clive Osmond from the MRC Environmental Epidemiology Unit, Southampton General Hospital were the first to associate variation in developmental environment, particularly of nutrients leading to low birth weight babies, and health outcomes 50–60 years later^(36,46)^. This caused a paradigm shift in how scientists and clinicians thought about determinants of health. Nevertheless, the effect size was, and remains, very small, requiring epidemiological sample sizes to see relatively small average shifts from the mean of blood pressure (cf. hypertension) or blood glucose (cf. type 2 diabetes). For populations, this shift in effect size is important; for any individual, less so. Where are we now?

At a population level, many epidemiological studies have documented the presumed consequences of ‘developmental programming’. In India, industrial and economic growth are closely paralleled by increasing CHD and type 2 diabetes, largely due to a ‘thin-fat’ nutrition transition (developmental thrift ‘thin’ to postnatal excess ‘fat’)^(47)^. In countries where entire groups of individuals or populations were exposed to famine, the adult offspring of those women exposed while pregnant, but not after, have increased risk of many non-communicable diseases^(48–50)^. At the level of an individual born small-for-gestational-age, what biological read-outs are there? Recent evidence suggests consistent, epigenetic alteration to single genes in the liver may increase the risk of metabolic dysfunction; in humans, the retinoid X receptor-*α* was identified^(51)^, whereas in rodents it was hepatocyte nuclear factor 4-*α*^(52)^. The authors propose that perinatal epigenetic analysis may have utility in identifying individual vulnerability to later obesity and metabolic disease. Will the next few decades see such ‘epigenetic screening’? Perhaps it could be realistic if applied in a targeted fashion, with follow-up nutritional advice.

In 2014, we developed a research excellence framework impact case study, ‘*Influencing national and international health policies regarding a role for early life nutrition on the risk of non-communicable disease in adulthood*’ and it was challenging to document tangible, demonstrable ‘impact’. Influential medical associations such as the British Medical Association had produced guidance for women of reproductive age, ‘*Early Life Nutrition and Lifelong Health*’ in 2009. Two years later, the Department of Health commissioned a Scientific Advisory Committee on Nutrition report on ‘*The influence of maternal, fetal and child nutrition on the development of chronic disease in later life*’, with a remit to identify opportunities for nutritional intervention that could influence the risk of chronic disease in later life in the offspring. They concluded that ‘*the evidence associating early life nutrition with later risk of chronic disease is variable in quality. Most of the human evidence demonstrates associations that are susceptible to confounding by environmental and behavioural factors at different stages of the life course… although markers of later risk, particularly if validated in animal models, maybe useful’.* The Scientific Advisory Committee on Nutrition made six public health recommendations to the Department of Health, including a recommended strategy to promote, protect and support breast-feeding, and to optimise the diets and body composition of young women. The ‘Change4life’ campaign was developed by the British Government and adopted into NHS online literature (Healthier Families – Home – NHS (www.NHS.uk)). On the international stage, a recent WHO/UNICEF report highlighted the importance of maternal nutrition during preconception, during pregnancy, the importance of lactation and early childhood nutrition^(53)^. There is no doubt that the DOHaD concept and related research have reached many more people than before Barker and Osmond first published their data. More recently, social media is undoubtedly increasing their message and promoting the DOHaD message to the public e.g. Twitter @DOHAD, https://imprintedlegacy.com. However, clear strategies to implement the research findings into clinical practice and policy change have been somewhat lacking.

In a recent leading-edge review of the topic, Stephenson *et al* report that while several studies show that micronutrient supplementation during pregnancy can be used to correct important maternal nutrient deficiencies – if they have been identified – effects on child health outcomes are disappointing^(54)^. The authors go on to say that ‘*Other interventions to improve diet during pregnancy have had little effect on maternal and newborn health outcomes*’. They acknowledge that the key period for intervention (diet and/or lifestyle) is peri-conception, and few interventions have been designed for implementation at this time, despite much research emphasising this critical period^(55–58)^. The authors conclude that ‘*a sharper focus on intervention before conception is needed to improve maternal and child health and reduce the growing burden of non-communicable diseases*. *Alongside …efforts to reduce smoking, alcohol consumption, and obesity in the population, we call for heightened awareness of preconception health, particularly regarding diet and nutrition*^(54)^’.

Perhaps the greatest impact of DOHaD science over the last few decades, therefore, is the pervading influence it now has on all aspects of biological phenomena, whether a result of current events such as the COVID-19 pandemic, the ongoing Ukrainian-Russian conflict or past famines (e.g. Dutch hunger winter, Siege of Leningrad) or atrocities (e.g. the Holocaust). As a direct result of DOHaD, the questions that are now asked include consideration of what lasting intergenerational impacts those children gestated or born during such times might carry forward into their adult lives, their lifelong ‘imprint’ of their mother’s experience with further, potentially heritable, consequences for their own children. This concept was explored by Sarah Richardson in a recent book, ‘*The Maternal Imprint: The Contested Science of Maternal-Fetal Effects*’^(59)^. Mentioned in the book is the seminal study of Yehuda, who concluded that Holocaust survivors have, ‘*an intergenerational epigenetic priming of the physiological response to stress in offspring of highly traumatized individuals’*^(60)^. From a personal perspective, Richardson nicely summarises the paradigm of DOHaD as the ‘*long reach of the womb [to be] at once beguiling, challenging to validate, stubbornly persistent once launched, and beset by scientific controversy*’ and suggests caution when interpreting DOHaD at the level of the individual – because, by definition, the politics of behavioural change in the context of DOHaD are highly gendered. Mothers are more often held responsible for the outcomes of pregnancy.

Over the last decade, however, a wealth of data has arisen regarding an independent role for the *paternal* environment on offspring outcomes^(61–65)^. Thus, when we think about the ‘developmental environment’, rather than a myopic focus on the mother, it is far more appropriate to target the broader environment when considering any possible interventions, that is, the maternal/paternal environment, their dietary pattern, their social and behavioural environment. For the impact of DOHaD science to be realised, it is towards these factors that ameliorative efforts should be targeted. Mendelian randomisation as a novel analytic method to ascribe relative importance of transgenerational effects to either the mother, father or both^(66)^ suggested that while the maternal environment had the greater effect, there was significant variance associated with the father^(67)^. In a recent letter, Merino and Tobias reiterated how ‘causal relationships’ between the nutrition of the mother (and father, assuming a nuclear family environment) and delayed developmental outcomes in the progeny are notoriously difficult to determine from observational studies, due to extensive confounding from unforeseen environmental factors^(68)^. Even with the more robust Mendelian randomisation studies, potential confounding can arise as diets may vary across time, substitution of one macronutrient by definition means another is also altered. Which is correlated with the measure of health? Furthermore, health outcomes may be correlated with unforeseen and unmeasured psychosocial, behavioural or environmental factors; the ‘Mediterranean Diet’ is considered a healthy option but is the effect size similar when consumed outside of the Mediterranean or does the additional sun and cultural environment experienced in the Mediterranean mean ‘the Diet in the Mediterranean’ is healthier.

## Development and the art of nutritional maintenance

### Quality of nutrition (not quantity)

Restriction of specific dietary components has a much greater impact on outcomes in offspring than global restriction of energy. We have directly tested this hypothesis in sheep (e.g. total energy^(69)^ *v*. low protein only^(70)^) and can report much greater treatment effects of macronutrient, as opposed to global energy, restriction. Further studies involving restriction of only one-carbon substrates, in sheep, also reiterated this point^(58)^. In rodents, the low protein model of developmental programming is widely published. Normal protein levels in rat chow have been set at approximately 19 %^(71)^ of total weight (21 % casein) and commonly used restricted levels range from 12 % (mild)^(72)^ to 6 % (severe)^(73)^ of total energetic intake. However, many of these diets are formulated using only semi-purified ingredients, meaning that while ‘low protein’ is the intervention applied, the maternal and fetal experience is of low protein plus one of higher sucrose, glucose, starch or another ingredient. For example, in a balanced rat diet, sucrose would normally only make up 5–6 % of total energetic intake; in many low protein diets sucrose may range from 21 %^(74)^ to 66 %^(75)^. The additional simple sugars could confound the effects of the low protein diet. In other experimental (rodent) studies, where nutrition is the treatment applied, such as the paradigm of a high fat, high sugar (or ‘junk-food diet’), the control groups for comparison were more often than not given laboratory chow, which varied in many more nutrients than fat and sugar, that is, outcomes were based on treatment effects of two completely different diets, a practice widely discredited^(76)^. For example, in one study, two different companies produced two different diets where all three macronutrients varied between diets, and a further six more differences were apparent (e.g. inclusion of Na, K and methionine were all different in the high-fat diet, cf. control diet)^(77)^.

We, therefore, argue that many of the problems associated with repeatability and reliability in scientific studies where nutrition has been used as a treatment could be ameliorated if greater attention was paid to the quality of the diets, using purified ingredients rather than semi-purified, for example^(78–80)^ and not comparing laboratory ‘chow’ to a given other diet. To what extent does laboratory chow vary between companies, countries or institutions? Indeed, Kozul *et al* found that feeding different laboratory chows *per se* significantly affected gene expression in two different mouse organs^(81)^. The type of diet used in laboratory studies should have as much focus and thought as the choice of strain, age, techniques and statistics^(82)^.

### Nutrition is personalised

Perhaps not remarkably, when we consider any adult individual is a product of their developmental experience, which even in twins is variable, a recent study (*n* 1002 twins) demonstrated widely variable responses to the same standardised meal. For example, standardised deviation (sd) in postprandial blood TAG between individuals was 103 %, for glucose the sd was 68 % and for insulin the sd was 59 %^(83)^. Indeed person-specific factors, such as gut microbiome, had a greater influence (7·1 % of variance) than did meal macronutrients (3·6 % of variance) for postprandial lipaemia, but not for postprandial glycaemia (6·0 and 15·4 %, respectively). Similar conclusions were drawn from another recent study that characterised serum metabolites with the colonic microbiome in individuals with CHD – the condition had a personalised risk profile that was likely driven by variation in diet^(84)^. Since, food intake, particularly of (in)soluble fibre, largely influences the composition of the microbiome^(85)^, in the context of DoHAD, characterising Gene × Environment (G × E) interactions, where environment also includes the diversity of current food intake and the microbiome, is perhaps more important than characterising the mean physiological adaptive response *per se*. For example, those individuals moving from nutritional paucity to nutritional abundance may experience a degree of the thin-fat syndrome and central adiposity, but it is probably more important to characterise individual variability within groups – the outliers – in order to understand mechanistic responses driven by DOHaD.

### Dietary fibre is important

Much of the previous discussion has been around either variation in macronutrients in experimental studies or certain dietary factors being associated with disease (e.g., high fat, high sugar). Comparatively few studies have directly considered the one macronutrient that often varies in parallel – NSP or fibre. A basic google scholar search for ‘low protein diet, chronic disease’ gave 3 350 000 results *v*. 260 000 for ‘low fibre diet, chronic disease’ (accessed 22/02/22). There is a paucity of experimental studies directly investigating dietary fibre in the context of long-term health, despite the recent discovery of the gut microbiome and links to non-communicable disease states such as obesity^(86)^. A recent meta-analysis of studies examining carbohydrate quality and human health concluded that relatively high intakes of dietary fibre and whole grains were complementary regarding the striking beneficial dose–response effect of replacing refined grains with whole grains^(87)^. Pro- and pre-biotics targeting the gut microbiome are widely available, but it is far more important to eat foods containing them (and thus other important micronutrients) than to take them as a supplement^(88)^. Indeed, a theoretical fibre enrichment intervention (2·2 g/d increase from baseline fibre intake in the UK of 19·9 g/d) suggests that 5·9 % of subjects could achieve a weight reduction, 72·2 % a reduction in cardiovascular risk and 71·7 % a reduced risk of type 2 diabetes risk with fibre fortification^(89)^. Food as a means to support long-term health should emphasise the beneficial effects a relatively high-fibre diet can have, often greater than many medical interventions, rather than over-emphasise what should not be eaten (soft drinks, added sugar, salt).

To conclude, the concept of ‘*Quality’* was considered undefinable by Robert Persig in his book, ‘*Zen and the Art of Motorcycle Maintenance*. We hope that quality of nutrition science for ‘nutritional maintenance’ is able to be defined and is not an ‘Art’; quality nutritional maintenance is a relatively simple concept; the focus should be upon intake of relatively unprocessed and unrefined foods with high-quality proteins and fats, ample fibre and lots of coloured, seasonal foods which deliver lots of additional micronutrients and pre/pro-biotics to support gastrointestinal health. Translating these concepts to a poor-quality developmental experience married with an actionable outcome to ameliorate the proposed ‘DOHaD’ phenotype is the real challenge yet to be defined.
